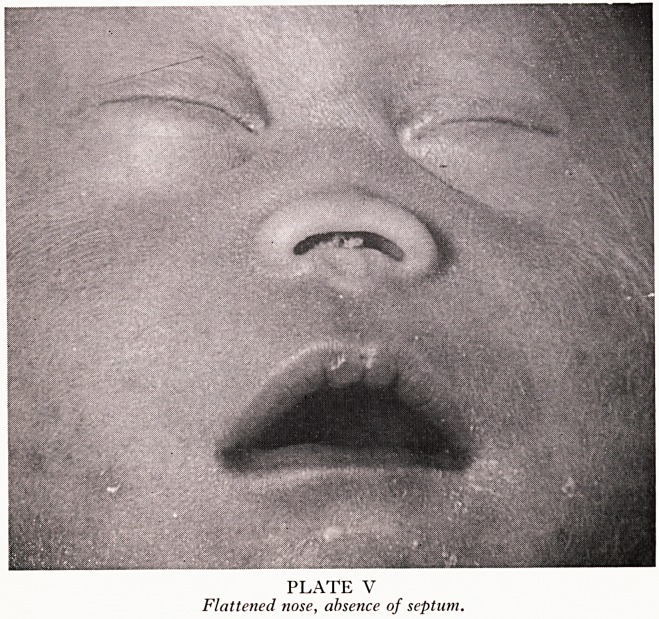# Arhinencephaly—A Short Review

**Published:** 1965-01

**Authors:** T. F. Draisey, S. A. Cullen, Sheila A. Faint

**Affiliations:** Department of Pathology, Southmead Hospital, Bristol; Department of Pathology, Southmead Hospital, Bristol; Department of Pathology, Southmead Hospital, Bristol


PLATE IV
*Brain: absence of olfactory tracts.*

PLATE V
*Flattened nose, absence of septum.*

### ARHINENCEPHALY—A SHORT REVIEW

BY

T. F. DRAISEY, S. A. CULLEN, AND SHEILA A. FAINT

*Department of Pathology, Southmead Hospital, Bristol*

Abnormalities of the central nervous system form the largest group of congenital defects causing perinatal mortality (Butler and Bonham, 1963). Malformation of the rhinencephalon is one of the lesser known abnormalities of the brain, but it occurs with sufficient frequency for it to be borne in mind by the pathologist who is concerned with autopsies upon newly born infants.

The rhinencephalon or "smell brain" is not very prominent in the human and it is mostly tucked away in the midline, hidden by the cerebral hemispheres. It is usually divided into two parts; the afferent part is composed of the olfactory bulbs and tracts, the anterior perforated substance, the induseum griseum, and the hippocampal gyrus; while the fornix and the nucleus habenulae form the efferent pathway. The septum lucidum and part of the corpus callosum are also included within the rhinencephalon but their functions are not understood.

The term arhinencephaly was coined by Hans Kundrat in 1882. His syndrome consisted of prosorhinal malformations (e.g. hare lip, cleft palate and microphthalmia), absence of olfactory bulbs and tracts, and failure of cleavage of cerebral hemispheres which thus form a single "sphere" with a single cavity instead of two hemispheres with symmetrical cavities. He considered that the absence of the olfactory bulbs and tracts was the primary abnormality (Kundrat 1882).

Pedantically speaking the term arhinencephaly is a misnomer as it implies total agenesis of the rhinencephalon, and this is not the case because part (the prepiriform and hippocompal formations) is always present. (Stewart 1939, Yakovlev 1959). We shall use the term, however, to include all cases with absence of olfactory bulbs and tracts.

Arhinencephaly is usually associated with abnormal facies and with visceral and skeletal malformations. Abnormalities of the eyes are common, especially ocular fusion. This produces the cyclops deformity. Recently interest has been focused upon the chromosome pattern in arhinencephaly and trisomy of the 13–15 group has been recorded.

In this article we should like to describe six cases of arhinencephaly found at routine post-mortem examination of newly born infants during the past 2½ years. In this period nearly 600 autopsies were performed upon infants dying in the perinatal period.

### CASE HISTORIES

*Case 1*

The female second child of a woman with one normal child. The brain showed absence of the olfactory bulbs, olfactory tracts, anterior perforated substance, corpora mamillaria and septum lucidum. The corpus callosum, hippocampus, and fornix were normal.

*Case 2*

The female fourth child of a 23 year old woman who had had two normal children and an anencephalic stillbirth. The face was a typical cyclops. The brain was a macrogyric, simply convoluted "sphere" with a single ventricular cavity crowning a rudimentary corpus striatum and thalamus. There was no inter-hemispheric fissure and the corpus callosum, fornix, and septum lucidum were absent. The mamillary bodies, anterior perforated substance and pituitary stalk were all absent. The olfactory bulbs and tracts were absent. All the other cranial nerves were present and appeared to follow normal courses.

TABLE 1

| Case No | Type of Arhinencephaly | Associated Abnormalities | | | | | |
|---|---|---|---|---|---|---|---|
| | | C.N.S. | Ocular | Skull | Facial (all have abnormal noses) | Skeletal | Visceral |
| 1 | Minor | Occipital meningocoele | None | Occipital defect | Cleft palate | None | Polycystic kidneys |
| 2 | Major | Iniencephaly Meningomyelocoele | Scleral fusion | Absent cribriform plate and crista galli, orbital fusion | None | Spina bifida | None |
| 3 | Minor | None | Microphthalmia | None | Cleft palate | None | Coarctation of aorta. Single umbilical artery Patent vitello-intestinal duct Thyroglossal cyst Patent urachus Reduplication of right renal pelvis and ureter |
| 4 | Major | Hydrocephalus | None | Absent cribriform plate and crista galli | Absent nasal septum | None | None |
| 5 | Minor | None | None | Brachycephaly | None | None | None |
| 6 | Major | Microgyria | None | Microcephaly | Cleft palate Micrognathos | Polydactyly | Pulmonary atresia Ventricular and atrial septal defects Persistent vitello-intestinal duct |

*Case 3*

The male eighth child of a 43 year old woman with seven normal children.

The brain showed absence of olfactory bulbs, olfactory tracts, corpora mamillaria and anterior perforated substance. The corpus callosum showed absence of the splenium; the induseum griseum and septum lucidum were absent. The fornix, cingulate gyrus and hippocampus were all normal. The temporal lobes were rounded and the uncus was prominent. The rhinal sulcus was present.

*Case 4*

A male child dying aged 2 months.

The brain was hydrocephalic. The cerebral hemispheres were not separated, and superiorly consisted of a translucent sac of cerebrospinal fluid formed by dilatation of the common ventricle. There was no interhemispheric fissure and the corpus callosum, fornix, and septum lucidum were absent. The mamillary bodies, anterior perforated substance and pituitary stalk were all absent. The olfactory bulbs and tracts were absent. All the other cranial nerves were present and appeared to follow normal courses.

*Case 5*

A female second child of a 33 year old woman with one normal child.

The brain showed absence of the olfactory bulbs, olfactory tracts, anterior perforated substance, mamillary bodies and septum lucidum. The corpus callosum, hippocampus, and fornix were normal.

*Case 6*

The female fifth child of a 39 year old woman with four normal children.

The brain was microcephalic. There was absence of the olfactory bulbs, olfactory tracts, anterior perforated substance and mamillary bodies. The corpus callosum, fornix and septum lucidum were absent and the ventricular cavity was closed superiorly by interdigitating gyri. The cingulate gyrus was absent. The hippocampus was normal.

### DISCUSSION

These cases fall into two groups. In the first group (cases 2, 4, and 6) there was a major abnormality of the brain, with an absent corpus callosum. One infant (case 2) was a cyclops monster and the other two had most of the cebocephalic anomalies. In the second group (cases 1, 3, and 5) the cerebral abnormality was limited to absence of the olfactory bulbs and tracts and other parts of the afferent pathway (Plate IV)—this we call the minor deformity. These findings agree with those of Schwalbe and Josephy (1913). The various abnormalities are summarized in Table 1.

All forms of arhinencephaly are accompanied by facial abnormalities. These vary from the cyclops deformity (where, in contrast to the mythical Polyphemus, the nose is above the single eye) and the septumless nose of the cebocephalic (Plate V) to the minor form where the nose is only flattened.

Arhinencephaly is frequently accompanied by other abnormalities; cleft palate, ocular, cardiac and renal abnormalities seem to be common. Some workers report polydactyly and genital malformation. Absence of the cribriform plate and crista galli seems only to occur in the most severe forms.

Table 2 shows the incidence of arhinencephaly over $2\frac{1}{2}$ years compared with two well known abnormalities, congenital polycystic kidneys and atresia of the bile ducts. In this small series arhinencephaly occurs with at least the same frequency as congenital polycystic kidneys.

TABLE 2

Relative Frequency of Arhinencephaly

| | |
|---|---|
| Total Perinatal Autopsies .. .. | 594 |
| Total with Congenital Abnormality .. | 133 |
| Arhirencephaly .. .. .. | 6 |
| Polycystic Kidneys .. .. .. | 6 |
| Atresia of Bile Ducts .. .. .. | 1 |

Chromosomal abnormality associated with arhinencephaly has been described by Miller (1962) and Laurence (1964) who found trisomy of the 13–15 group. Each of their patients had a cleft palate and there were other major visceral abnormalities. Other cases of 13–15 trisomy have been reviewed by Lubs and his colleagues (1961) but arhinencephaly was not described. The only one of our patients to have chromosomal analysis performed was case 5, because of a peculiarly flat nose. This showed a normal karyotype. At autopsy, there were no visceral or skeletal abnormalities; only minor arhinencephaly. It may be that for the production of this minor deformity there is no need for gross chromosomal change.

*Acknowledgements*

We should like to thank Dr. F. J. W. Lewis and Dr. N. J. Brown for reading this paper and for their valuable criticism. We wish also to acknowledge the Ethel Showering Fund. Mr. W. G. Sweet kindly did the photography.

### REFERENCES

Bulter, N. R., and Bonham, D. G. (1963). *Perinatal Mortality*, Edinburgh.
Kundrat, H. (1882). *Wien. Med. Bl.*, **5**, 1395.
Laurence, K. M. (1964). *Arch. Dis. Child.*, **39**, 302.
Lubs, H. A. *et al.* (1961). *Lancet*, **ii**, 1001.
Miller, J. Q. *et al.* (1962). *Am. J. Dis. Child*, **104**, 532.
Schwalbe, E., and Josephy, H. (1913). *Morphologie der Missbildungen des Menschen und der Tiere*, part 3, Sec. 2.
Stewart, R. M. (1939). *J. Neurol. Psychati.* **2**, 303.
Yakovlev, P. I. (1959). *J. Neuropath. exp. Neurol.* **18**, 22.

## Figures and Tables

**PLATE IV f1:**
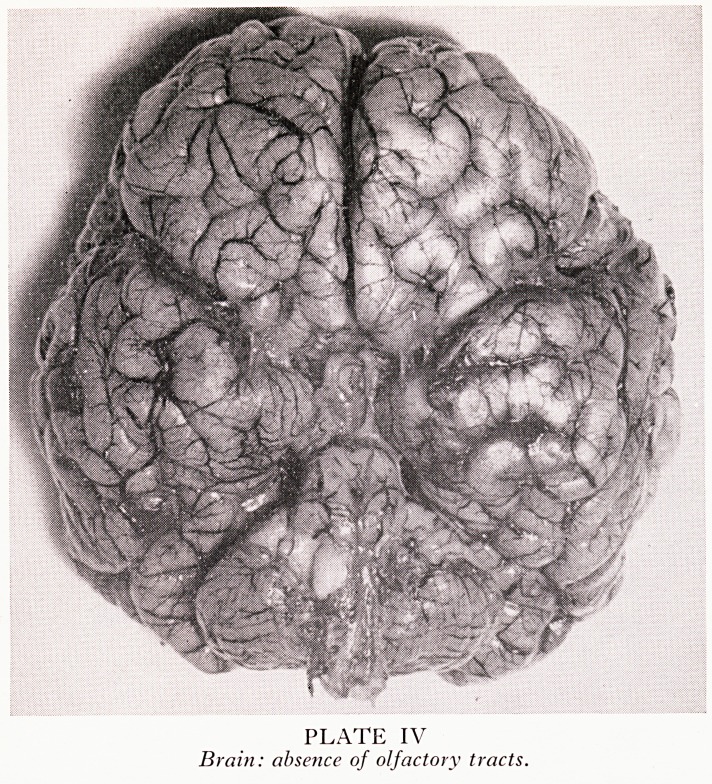


**PLATE V f2:**